# Novel Modulation Method for Multidirectional Matrix Converter

**DOI:** 10.1155/2014/645734

**Published:** 2014-09-14

**Authors:** Saman Toosi, Norhisam Misron, Tsuyoshi Hanamoto, Ishak Bin Aris, Mohd Amran Mohd Radzi, Hiroaki Yamada

**Affiliations:** ^1^Department of Electrical & Electronic, Faculty of Engineering, Universiti Putra Malaysia (UPM), 43400 Serdang, Selangor, Malaysia; ^2^Institute of Advanced Technology (ITMA), Universiti Putra Malaysia (UPM), 43400 Serdang, Selangor, Malaysia; ^3^Department of Biological Functions Engineering, Graduate School of Life Science and Systems Engineering, Kyushu Institute of Technology, 2-4 Hibikino Wakamatsu-ku, Kitakyushu 808-0916, Japan; ^4^Graduate School of Science and Engineering, Yamaguchi University, 2-16-1 Tokiwadai, Ube-shi, Yamaguchi 755-8611, Japan

## Abstract

This study presents a new modulation method for multidirectional matrix converter (MDMC), based on the direct duty ratio pulse width modulation (DDPWM). In this study, a new structure of MDMC has been proposed to control the power flow direction through the stand-alone battery based system and hybrid vehicle. The modulation method acts based on the average voltage over one switching period concept. Therefore, in order to determine the duty ratio for each switch, the instantaneous input voltages are captured and compared with triangular waveform continuously. By selecting the proper switching pattern and changing the slope of the carriers, the sinusoidal input current can be synthesized with high power factor and desired output voltage. The proposed system increases the discharging time of the battery by injecting the power to the system from the generator and battery at the same time. Thus, it makes the battery life longer and saves more energy. This paper also derived necessary equation for proposed modulation method as well as detail of analysis and modulation algorithm. The theoretical and modulation concepts presented have been verified in MATLAB simulation.

## 1. Introduction

More than 1.3 billion people in the world are not connected to a national grid. Although extension of the conventional electricity grid remains preferable mode of electrification, it is not economical for areas where the grid extension is difficult. Currently the stand-alone power system (SAPS) supplies local villages or individual users with lack access to electricity. Typical SAPS may be powered by one or more methods such as microhydroturbine, wind turbine, solar panel geothermal source, and diesel or biofuel generator to generate the electricity [1].

A major requirement for stand-alone power system is to ensure continuous power flow by storing excess energy from the energy sources. For example, hybrid systems with battery storage are employed as an efficient and reliable stand-alone system for remote areas [2]. Battery based systems (BBS) are amongst the SAPS models which a battery may employ in series or parallel with renewable energy source. In battery based systems, the input power of the system converts to desirable voltage and frequency through power electronic converters in order to supply the system loads and charge the battery [3, 4].

In recent years, the matrix converter becomes popular in the category of AC to AC converters due to the desirable features such as sinusoidal input and output current, generation of load voltage with arbitrary amplitude and frequency, and ability to control input power factor for any load [5]. In the early 1980s Venturini and Alesina proposed the principle of MC control [6]. They derived duty ratio functions that can be modulated by carrier signal. In this method, the voltage transfer ratio was limited to 0.5. Alesina and Venturini (1981) theoretically proved that the maximum voltage ratio, *q*_max⁡_, is equal to 0.866 for the three-phase MC when using balanced input voltage [7]. In 1989, Alesina and Venturini extended the voltage ratio from 0.5 to 0.866 by taking advantage of third harmonic injection methods [8].

The “indirect transfer function” was derived by Rodriguez in 1983 [9]. In this method, the matrix converter was described as virtual configuration of pulse with modulation (PWM) rectifier and inverter with “fictitious dc link.” The operational and technological research on MC were continued in different areas such as new topology of MC [10–13], input filter design [14, 15], unbalance operational conditions [16–18], safe and practical commutation strategies [10, 19], new control methods [20, 21], new modulation methods [22, 23], and new application such as hybrid vehicles [24].

Yoon and Sul (2006) [23] proposed new carrier based modulation methods for conventional matrix converter. This method is the same as conventional space vector pulse modulation (SVPWM) which is used in voltage source inverter. Yoon and Sul synthesized the sinusoidal input current with unity power factor by changing the slope of carrier and the proper offset voltage. The reference output voltages are calculated and compared with a discontinuous carrier to generate the gating signals. However, it is difficult to intuitively understand the modulation principle since it employs the offset for references and discontinues carrier signal. Furthermore, this method cannot be used for the MC typologies with a neutral connection.

The preliminary concepts of a new carrier based PWM strategy, named direct duty ratio PWM (DDPWM), are presented by Li et al. (2008). This method can be implemented without complex calculations and lookup tables and does not require the reference offset voltage. Based on the average value of each output phase in one switching period, the duty ratio values may be updated at each switching cycle by employing input phase voltages. Thus, the PWM signals are generated by comparison of these duty ratio values with a continuous triangular carrier waveform. The *q*_max⁡_ of 0.866 also can be easily obtained in the three-phase system by applying the third harmonic injection method to the output voltage references. Furthermore, the input power factor can be controlled by changing the slope of the carrier while maintaining the sinusoidal input currents [22]. Li and Choi (2009) extended the DDPWM to various topologies of matrix converter and derived the control schemes for alternative structures such as single-phase and three-phase four-leg matrix converters [25].

Multidirectional converter has recently been proposed as an alternative power conversion concept which has both rectifier and inverter capability [26, 27]. Most desired features of multidirectional converter can be fulfilled by using Matrix Converter structures. In the MDMC, a bidirectional switch is used, coupled between the power source and load, to provide both AC and DC properties, which cannot be achieved with conventional converters. This converter has ability to control the power flow and synthesise the desired output voltage by developing the space vector pulse width modulation (SVPWM) methods. In the SVPWM method, the modulation task of the multidirectional matrix converter can be resolved into the different imaginary stages of transformation including inverter and rectifier stage which are linked together by an imaginary DC link. However, the MDMC is not being able to inject power from generator and battery at the same time, since several vectors are utilized in one switching period [28]. Previous studies [29–31] show that, in conventional battery based system, generator should be disconnected from system when it is not being able to supply the demand power.

Based on the literature highlighted above, this study aims to inject power from battery and generator at the same time by changing the MDMC structure and increasing the number of time intervals of direct duty pulse width modulation method. In this study, the proposed modulation method determines the switching state of each output phase by employing the input DC phase voltages and input AC phase voltages based on per-output-phase average concept over one switching period. At the first step of each switching period, in order to generate the corresponding PWM signals, the duty ratio values for each output phase were calculated and the results compared the continuous triangular waveform. This new topology and new modulation method can increase the discharging time of battery in the battery based systems when the discharging time is directly proportional to the generator output voltage. Therefore, the multidirectional matrix converter with a new modulation method is expected to breakthrough towards new technological advancements in the area of sustainable energy and power electronics.

## 2. Proposed MDMC Structure for Battery Based System 

**Figure 1:**
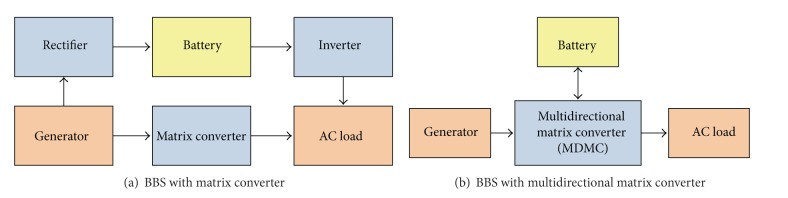
Block diagram of system with parallel battery connection.

Batteries are not efficient as a whole. Some energy is lost as heat and chemical reactions when charging and discharging. In common, the lead acid battery's efficiency is around 85% when state of charge (SOC) is varied from 0 to 100% [32]. In battery based stand-alone power system (BBSAPS), when the battery is connected in series, total electricity generation from system will be stored in battery before transmitting to the loads, while in system with parallel battery connection only the excess electricity will be saved in battery. Hence, a system with parallel battery connection is more efficient compared to a system with series battery connection. In addition, the parallel battery based system can be modified by combining all converters as a single converter which is indicated as multidirectional matrix converter.  Figure 1 shows the comparison of block diagram of BBSs with matrix converter and the proposed MDMC system.

**Figure 2:**
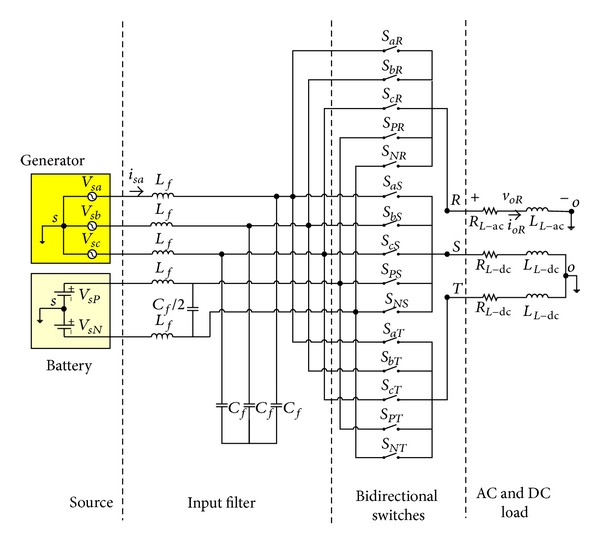
Multidirectional matrix converter circuit.

The multidirectional matrix converter is a single-stage converter which has a *m* × *n* matrix (or array) of bidirectional power switches to connect an *m*-phase voltage source to an *n*-phase load directly. In general, the proposed MDMC needs 15 bidirectional switches that is one switch between each input and output phases.  Figure 2 shows the circuit configuration of proposed MDMC including the positive DC input voltage (battery), negative DC input voltage (battery), three-phase input voltages (AC generator), multidirectional matrix converter and resistor-inductor (*R*-*L*) load, and second-order *L*-*C* filter which is used at the input terminals to filter out the high frequency harmonics of the input currents. In this study the *R*_*L*-dc_ and *L*_*L*-dc_ and the *R*_*L*-ac_ and *L*_*L*-ac_ are considered as DC and AC load, respectively.

In the MDMC, the switching method should have sinusoidal waveforms at the arbitrary magnitude, frequency in AC side, and clean DC voltage at the DC side. The input currents also should be sinusoidal at the desired power factor. In order to achieve this target, a proper switching pattern should be applied to the switches of the MDMC in each switching period. The general switching function for the switches of the MDMC can be described as follows:

(1)   Sij(t)={1,Sij  closed,0,Sij  open,  i=a,b,c,P,N j=R,S,T,

where the *S*_*ij*_ refers to the switch on input line “*i*” and output line “*j*.”

Moreover, input phases should not be short circuited and output phases should never be opened due to the inductive nature of typical loads. In this study, these two constraints can be expressed as below:

(2) Saj+Sbj+Scj+SPj+SNj=1, j={R,S,T}.

By considering two states for each switch in (1) and by applying the limitation of (2) to the switching algorithms of the proposed MDMC, allowable combinations will be derived based on the DDPWM technique. Voltages and currents of sources and voltages and currents of load in Figure 2 can be expressed as vectors that are defined by (3) where the *X* can be input and output phase-to-neutral voltage vectors or the MDMC input and output current vectors:

(3) $$\begin{array}{c} {\overset{-}{X}}_{RST}=\left[\begin{array}{c} X_{R}\left(t\right)\\ X_{S}\left(t\right)\\ X_{T}\left(t\right) \end{array}\right],\;\;{\overset{-}{X}}_{abcPN}=\left[\begin{array}{c} X_{a}\left(t\right)\\ X_{b}\left(t\right)\\ X_{c}\left(t\right)\\ X_{P}\left(t\right)\\ X_{N}\left(t\right) \end{array}\right]. \end{array}$$

The MDMC instantaneous switching function matrix can be expressed as follows:

(4) $$\begin{array}{c} S=\left[\begin{array}{ccccc} s_{aR} & s_{bR} & s_{cR} & s_{PR} & s_{NR}\\ s_{aS} & s_{bS} & s_{cS} & s_{PS} & s_{NS}\\ s_{aT} & s_{bT} & s_{cT} & s_{PT} & s_{NT} \end{array}\right]. \end{array}$$

Equation (5) shows the relation between load, input voltages, and currents, where *S* ^*T*^ is the transpose matrix of *S* matrix:

(5) $$\begin{array}{c} {\overset{-}{V}}_{RST}=S\cdot {\overset{-}{v}}_{abcPN},\;\;\;{\overset{-}{i}}_{abcPN}=S^{T}\cdot {\overset{-}{i}}_{RST}. \end{array}$$

**Figure 3:**
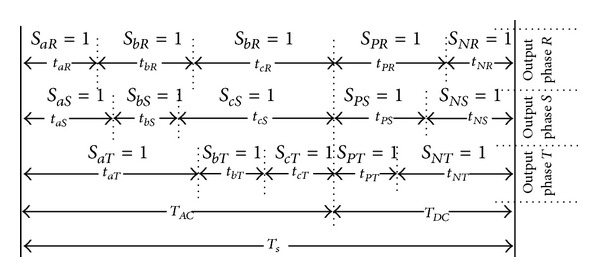
The switching pattern in a sequence period.

Modulation rules can be derived by applying the different switching pattern to the power switches (see Figure 3).

As indicated in Figure 3, the output phase “*R*” is connected to the input phase “*a*” during *t*_*aR*_ and when *T*_*s*_ is the sequence period of switching for MDMC system. It is also connected to phase “*b*,” “*c*,” “*P*,” and “*N*” during time periods *t*_*bR*_, *t*_*cR*_, *t*_*PR*_, *t*_*NR*_, respectively. Arbitrary amplitude and frequency can be generated by modulating the duty cycle of the switches using their respective switching functions.

If *d*_*ij*_(*t*) = *t*_*ij*_/*T*_seq_, the restrictions of the duty cycle (based on (2)) can be represented as below:

(6) 0≤dij≤1,  daj+dbj+dcj+dPj+dNj=1,              i∈(a,b,c,P,N), j∈(R,S,T).

The matrix *S* can be replaced by matrix *D*  (3 × 5) and finally the low frequency transfer matrix is defined as below:

(7) $$\begin{array}{c} \overset{-}{v_{RST}}=D\cdot \overset{-}{v_{abcPN}},\;\;\overset{-}{i_{abcPN}}=D^{T}\cdot \overset{-}{i_{RST}}, \end{array}$$

where *i*_*RST*_ and *v*_*RST*_ are a set of sinusoidal currents and arbitrary amplitude, frequency output voltages, and *v*_*abcPN*_ and *i*_*abcPN*_ are sinusoidal input voltages and input currents at the MDMC terminals.

## 3. Modulation Method 

In this modulation method, reference output phase voltage can be synthesized by utilizing all five input phase voltages over one switching period in the average sense. Therefore, the switching period *T*_*s*_ is divided into two time periods, *T*_*c*_ and *T*_3_. During *T*_*c*_, the input phases of AC generator are connected to a corresponding output terminal, and during *T*_3_ the input phases of DC battery are connected to a corresponding output terminal. In addition, the time interval *T*_*c*_ is divided into two periods *T*_1_ and *T*_2_. Also, the MX, MD, and MN denote the instantaneous values of maximum, medium, and minimum input voltages of AC generator. Furthermore, POS and NEG denote the instantaneous values of positive and negative input voltages of DC battery, respectively. During *T*_1_, the line-to-line voltage between MX and MN is used, which is the maximum line-to-line voltage among three line-to-line input voltages of generator at the sampling instant. During *T*_2_, the second maximum line-to-line voltage is used which is MX to MD for switching pattern-I and MD to MN for switching pattern-II. Finally, during *T*_3_ the line-to-line voltage between POS and NEG is employed.

**Figure 4:**
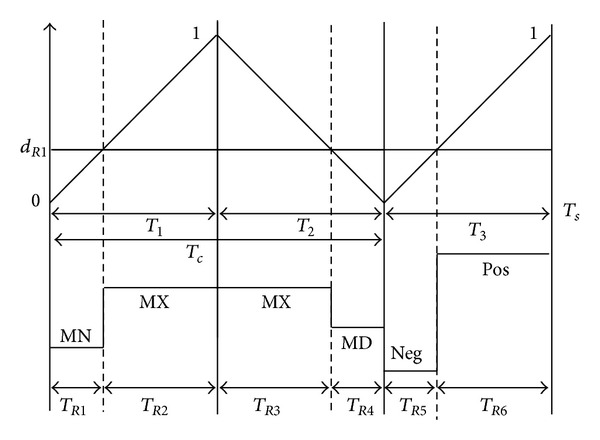
Output *R*-phase switching state in switching pattern-I.

In this method, the three line-to-line input voltages of the generator and the input voltages of batteries are read continuously at the sampling instant. Then, duty ratio values (range between 0 and 1) are predetermined for each output phase at the beginning of each switching period. Also, the duty ratio of each phase is compared with a common continuous triangular carrier waveform, in order to generate the corresponding six time subintervals (see Figure 4). These six time subintervals determine the connection time of the corresponding output terminal to the input phases during one switching cycle. Therefore, the desired output voltage can be synthesized by updating the duty ratio value during each switching period. In addition, the input power factor can be controlled by manipulating the slopes of the triangular carriers. Due to the time subintervals extension, this method is called extended direct duty pulse width modulation (EDDPWM).

### 3.1. Switching Pattern-I

Figure 4 shows the switching pattern-I, where the *R*-phase duty ratio value (*d*_*R*1_) is compared with triangular carrier waveform to generate the *R*-phase output voltage. The output phase is changed during the switching pattern-I from MN → MX → MX → MD → NEG → POS, consequently. The actual output voltage merge of *R*-phase is illustrated in Figure 6 when applying switching pattern-I. As illustrated in Figures 4 and 6, the output phase “*R*” is connected to the input phase “MN” during *T*_*R*1_ and when *T*_*s*_ is the sequence switching period. And it is connected to phases “MX,” “MX,” “MD,” “NEG,” and “POS” during time periods *T*_*R*2_, *T*_*R*3_, *T*_*R*4_, *T*_*R*5_, and *T*_*R*6_, respectively. These six time subintervals can be represented as (8), where *d*_*R*1_ is the *R*-phase duty ratio value and carrier slops are defined as *m* = *T*_1_/*T*_*c*_ and *n* = *T*_*c*_/*T*_*s*_:

(8) TR1=dR1mnTs,TR2=(1−dR1)mnTs,TR3=(1−dR1)(1−m)nTs,TR4=dR1(1−m)nTs,TR5=dR1(1−n)Ts,TR6=(1−dR1)(1−n)Ts.

The fluctuation of the input voltage is negligible during the switching periods. Thus, the integration of the output voltage *v*_*oR*_ over *T*_*s*_ can be expressed in

(9) ∫0TsvoR dt≅TR1·MN+(TR2+TR3)·MX +TR4MD+TR5·NEG+TR6·POS.

Based on (8) and (9), the average output voltage can be expressed in terms of *m* and *n* as follows:

(10) v−OR=1Ts∫0TsvoR dt≅dR1·(−(1−n)POS−n·MX+(1−m)·n·MD    + m·n·MN+(1−n)·NEG) +n·MX−(1−n)·POS.

For the present switching cycle, the duty ratio value, *d*_*R*1_, can be written as follows:

(11) dR1=(voR∗−n·MX−(1−n)·POS) ×(−(1−n)POS−n·MX+(1−m)·n·MD    + m·n·MN+(1−n)·NEG)−1,

where the *v*_oR_* is the *R*-phase output voltage command which is equal to the ${\overset{-}{v}}_{OR}$.

### 3.2. Switching Pattern-II

**Figure 5:**
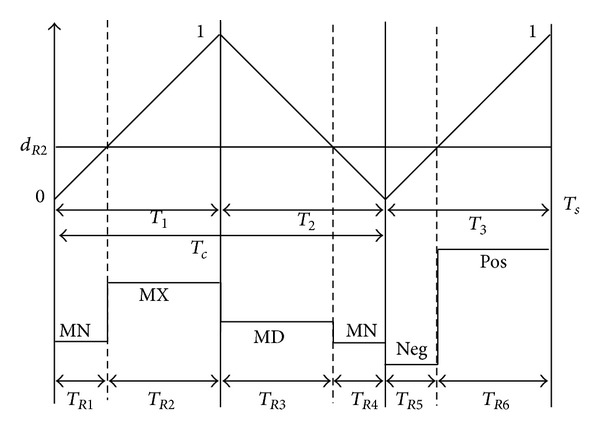
Output *R*-phase switching state in switching pattern-II.

The procedure to drive the equation for switching pattern II is the same as the previous switching pattern.  Figure 5 illustrates the case of switching pattern II where the *R*-phase duty ratio value (*d*_*R*2_) is compared with triangular carrier waveform to generate the *R*-phase output voltage. The output phase is changed during the switching pattern-II from MN → MX → MD → MN → NEG → POS, consequently. The actual output voltage merge of *R*-phase is illustrated in Figure 7 when the output phase “*R*” is connected to the input phases during the time subintervals sequentially. Similarly, the integration of the output voltage *v*_*oR*_ and the average output voltage ${\overset{-}{v}}_{OR}$ is presented as below:

(12) ∫0TsvoR dt≅(TR1+TR4)·MN +TR2·MX+TR3MD+TR5·NEG+TR6·POS,v−OR=1Ts∫0TsvoR dt≅dR2·(−(1−n)POS−mn·MX     −(1−m)·n·MD+n·MN     +(1−n)·NEG) +(1−n)·POS−m·n·MX+(1−m)·n·MD.

By letting the ${\overset{-}{v}}_{OR}$ be equal to *v*_oR_* the duty ratio value *d*_*R*2_ can be written as follows:

(13) dR2=(voR∗−n·MX−(1−n)·POS) ×(−(1−n)POS−n·MX+(1−m)·n·MD    + m·n·MN+(1−n)·NEG)−1.

### 3.3. Outputs Voltage Merged for MDMC

Five bidirectional switches are used for each output phase to apply the switching pattern-I and II. The POS and NEG input phase are always constant while the MX, MD, and MN are selected by instantaneous comparison of the AC input phases. When the switching state for output phase “*R*” is POS, NEG, MX, MD, or MN, the output phase “*R*” is connected to the input phase which the voltage is POS, NEG, MX, MD, or MN, respectively. This modulation control method can be applied to the MDMC as a modular structure for each phase where each output phase has the independent reference control signal. This reference control signal can be different in terms of frequency, waveform shape, and amplitude.

The duty ratio of phases *S* and *T* is indicated as *d*_*S*_ and *d*_*T*_ and can be derived in the same way of phase *R* by letting the ${\overset{-}{v}}_{OS}$ and ${\overset{-}{v}}_{OT}$ be equal to the *S* and *T* phase voltage command *v*_oS_* and *v*_oT_*, respectively. Duty ratio of phases can be expressed as follows:

(14) $$\begin{array}{c} d_{R}=\{\begin{array}{cc} \frac{v_{oR}^{*}-n\cdot \text{MX}-\left(1-n\right)\cdot \text{POS}}{-\left(1-n\right)\text{POS}-n\cdot \text{MX}+\left(1-m\right)\cdot n\cdot \text{MD}+m\cdot n\cdot \text{MN}+\left(1-n\right)\cdot \text{NEG}}, & \text{for}\;\text{Pattern-I},\\ \frac{v_{oR}^{*}-n\cdot \text{MX}-\left(1-n\right)\cdot \text{POS}}{-\left(1-n\right)\text{POS}-n\cdot \text{MX}+\left(1-m\right)\cdot n\cdot \text{MD}+m\cdot n\cdot \text{MN}+\left(1-n\right)\cdot \text{NEG}}, & \text{for}\;\text{pattern-II}, \end{array}\\ d_{S}=\{\begin{array}{cc} \frac{v_{oS}^{*}-n\cdot \text{MX}-\left(1-n\right)\cdot \text{POS}}{-\left(1-n\right)\text{POS}-n\cdot \text{MX}+\left(1-m\right)\cdot n\cdot \text{MD}+m\cdot n\cdot \text{MN}+\left(1-n\right)\cdot \text{NEG}}, & \text{for}\;\text{Pattern-I},\\ \frac{v_{oS}^{*}-n\cdot \text{MX}-\left(1-n\right)\cdot \text{POS}}{-\left(1-n\right)\text{POS}-n\cdot \text{MX}+\left(1-m\right)\cdot n\cdot \text{MD}+m\cdot n\cdot \text{MN}+\left(1-n\right)\cdot \text{NEG}}, & \text{for}\;\text{pattern-II}, \end{array}\\ d_{T}=\{\begin{array}{cc} \frac{v_{oT}^{*}-n\cdot \text{MX}-\left(1-n\right)\cdot \text{POS}}{-\left(1-n\right)\text{POS}-n\cdot \text{MX}+\left(1-m\right)\cdot n\cdot \text{MD}+m\cdot n\cdot \text{MN}+\left(1-n\right)\cdot \text{NEG}}, & \text{for}\;\text{Pattern-I},\\ \frac{v_{oT}^{*}-n\cdot \text{MX}-\left(1-n\right)\cdot \text{POS}}{-\left(1-n\right)\text{POS}-n\cdot \text{MX}+\left(1-m\right)\cdot n\cdot \text{MD}+m\cdot n\cdot \text{MN}+\left(1-n\right)\cdot \text{NEG}}, & \text{for}\;\text{pattern-II}. \end{array} \end{array}$$

In the proposed method, the output voltages have been well synthesised by using two out of five line-to-line input voltages during each switching period, while the input currents are distorted. In order to improve the input current quality and reduce the input currents distortion, five input phases conducted the current during each switching period.

### 3.4. Inputs Current Merged for MDMC

The *n* and *m* can properly be adjusted to reduce the input current distortion in (14). This current distortion can be reduced by controlling the input power factor which is directly depending on the slope of the triangular carrier. By maintaining the *T*_*s*_ at a constant value and adjusting the value of *n* and *m* to the desired value, the input current is synthesized. The output voltage waveform will not be disturbed since *n* and *m* are considered in the derivation of (14).

The output currents are almost constant during the switching cycles; thus the input current can be merged based on the PWM switching pattern. These six time subintervals for each phase can be expressed as follows:

(15) TX1=dXmnTs=dXT1,TX2=(1−dX)mnTs=(1−dX)T1,TX3=(1−dX)(1−m)nTs=(1−dX)T2,TX4=dX(1−m)nTs=dXT2,TX5=dX(1−n)Ts=dXT3,TX6=(1−dX)(1−n)Ts=(1−dX)T3,               X=R,S,T.

#### 3.4.1. Switching Pattern-I

**Figure 6:**
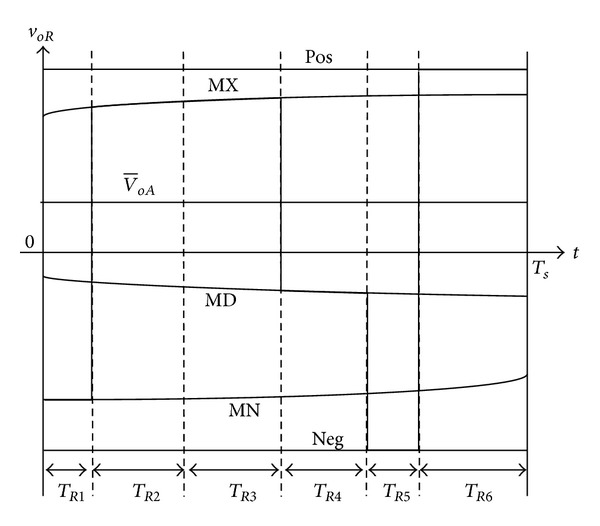
Output *R*-phase voltage synthesis in switching pattern-I.

Five inputs are connected to the output terminal through the bidirectional switches. According to the switching state as shown in Figures 4 and 6, the output phases *R*, *S*, and *T* during *T*_*R*1_, *T*_*S*1_, and *T*_*T*1_, are connected to the input phase whose voltage is MN. In the same way, the input phase MX is connected to the output phases *R*, *S*, and *T* during (*T*_*R*2_ + *T*_*R*3_), (*T*_*S*2_ + *T*_*S*3_), and (*T*_*T*2_ + *T*_*T*3_), respectively, and the input phase MD is connected to the output terminals *R*, *S*, and *T* during *T*_*R*4_, *T*_*S*4_, and *T*_*T*4_, respectively. In addition, the input phase NEG is connected to the output terminals *R*, *S*, and *T* during *T*_*R*5_, *T*_*S*5_, and *T*_*T*5_, respectively, and input phase POS is connected to the output terminals *R*, *S*, and *T* during *T*_*R*6_, *T*_*S*6_, and *T*_*T*6_, respectively. By applying the average concept to each input phase, the input current can be presented as follows:

(16) isMXTs=(TR2+TR3)ioR+(TS2+TS3)ioS+(TT2+TT3)ioT,isMDTs=TR4ioR+TS4ioS+TT4ioT,isMNTs=TR1ioR+TS1ioS+TT1ioT,isNEGTs=TR5ioR+TS5ioS+TT5ioT,isPOSTs=TR6ioR+TS6ioS+TT6ioT.

By substituting (15) into (16), the instants value of the input phase current during one switching cycle can be obtained as follows:

(17) isMXTs=−Tc·(dRioR+dSioS+dTioT),

(18) isMDTs=T2·(dRioR+dSioS+dTioT),

(19) isMNTs=T1·(dRioR+dSioS+dTioT),

(20) isNEGTs=T3·(dRioR+dSioS+dTioT),

(21) isPOSTs=−T3·(dRioR+dSioS+dTioT).

By substituting (19) into (17), the *m* can be obtained as follows:

(22) $$\begin{array}{c} m\equiv \frac{T_{1}}{T_{c}}=-\frac{i_{s\text{MN}}}{i_{s\text{MX}}}. \end{array}$$

Also, by calculating the *i*_*s*MX_ · *T*_*s*_ based on (*i*_*s*MX_ + *i*_*s*POS_) · *T*_*C*_, the *n* can be obtained as below:

(23) $$\begin{array}{c} n\equiv \frac{T_{c}}{T_{s}}=\frac{i_{s\text{MX}}}{\left(i_{s\text{MX}}+i_{s\text{POS}}\right)}. \end{array}$$

#### 3.4.2. Switching Pattern-II

**Figure 7:**
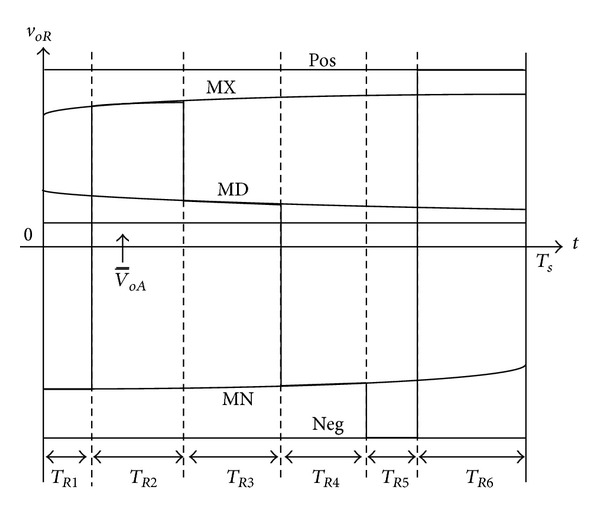
Output *R*-phase voltage synthesis in switching pattern-II.

Like switching pattern-I, by applying the switching pattern-II which is indicated in Figures 5 and 7, input phase MX is connected to the output phases *R*, *S*, *T* during time subinterval *T*_*R*2_, *T*_*S*2_, and *T*_*T*2_, respectively. Similarly, the input phase MD is connected to the output phases *R*, *S*, and *T* during *T*_*R*3_, *T*_*S*3_, and *T*_*T*3_, respectively and the input phase MN is connected to the output phases *R*, *S*, and *T* during (*T*_*R*1_ + *T*_*R*4_), (*T*_*S*1_ + *T*_*S*4_), and (*T*_*T*1_ + *T*_*T*4_), respectively. Finally, the input phase NEG is connected to the output terminals *R*, *S*, and *T* during *T*_*R*5_, *T*_*S*5_, and *T*_*T*5_, respectively, and input phase POS is connected to the output terminals *R*, *S*, and *T* during *T*_*R*6_, *T*_*S*6_, and *T*_*T*6_, respectively. The input currents can be presented as follows:

(24) isMXTs=TR2ioR+TS2ioS+TT2ioT,

(25) isMDTs=TR3ioR+TS3ioS+TT3ioT,

(26) isMNTs=(TR1+TR4)ioR+(TS1+TS4)ioS+(TT1+TT4)ioT,

(27) isNEGTs=TR5ioR+TS5ioS+TT5ioT,

(28) isPOSTs=TR6ioR+TS6ioS+TT6ioT.

By considering the time intervals for *T*_*s*_ and substituting (26) into (24), the *m* can be represented as follows:

(29) $$\begin{array}{c} m\equiv \frac{T_{1}}{T_{c}}=-\frac{i_{s\text{MX}}}{i_{s\text{MN}}}. \end{array}$$

In addition, by calculating the *i*_*s*MX_ · *T*_*s*_ based on (*i*_*s*MX_ + *i*_*s*POS_) · *T*_*C*_, the *n* can be obtained as below:

(30) $$\begin{array}{c} n\equiv \frac{T_{c}}{T_{s}}=\frac{i_{s\text{MN}}}{\left(i_{s\text{MN}}-i_{s\text{POS}}\right)}. \end{array}$$

Furthermore, when the power factor is one in balance system, the currents *i*_*s*MN_, *i*_*s*MX_, and *i*_*s*POS_ can be replaced by voltages *v*_*s*MN_, *v*_*s*MX_, and *v*_*s*POS_ (see (22), (23), (29), and (30)). As the input voltage is directly sensed from the power circuit, the modulation calculation becomes easier.

To achieve unity power factor, load can be assumed as current source in one switching cycle; accordingly, input current can be synthesized based on the switching state of the output phase current. The magnitude of each input current varied based on the ratio between *T*_1_ and *T*_2_ and ratio between *T*_*c*_ and *T*_3_ while *T*_*s*_ is constant. On the other hand, due to the missing of the energy storage component in MDMC, the input and output powers should be kept balanced all the time at any load. The practical selection of *n* and *m* for switching pattern-I and II can be determined by the input voltage angle *α*_*i*_, to synthesize sinusoidal input current.

**Figure 8:**
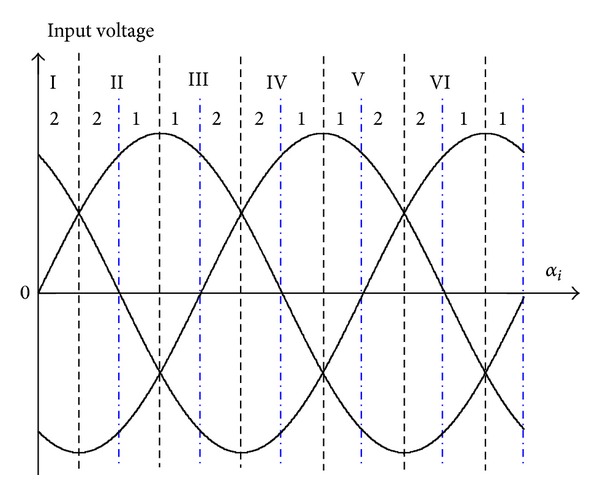
Interval voltage sector based on the input voltage (*α*_*i*_).

The three-phase input voltages signal shown in Figure 8 is divided into the 6 segments and each segment is divided into two sectors which correspond to either switching pattern-I or II. By letting the power factor of system equal to *φ*, the input current angle can be obtained as follows:

(31) $$\begin{array}{c} \beta_{i}=\alpha_{i}+\varphi . \end{array}$$

In the proposed modulation, input power factor is controlled by adjusting the value of *n* and *m* (Table 1). Based on the available maximum line-to-line voltage, the range of duty ratio is changed during time intervals *T*_1_ and *T*_2_. The range of available voltage ratio during the *T*_1_ is higher than *T*_2_. Therefore, the desired value of *m* is varied from 0.5 to 1 in each sector to reach the maximum power of input source. In Table 1, the *D* = *v*_*s*MX_/(*v*_*s*MX_ + *v*_*s*POS_) indicates the magnitude variation of *n*, where *v*_*s*MX_ is nominal value of generator input voltage.

## 4. Simulation Result

Simulation of the proposed modulation method for MDMC is performed by using MATLAB software. The system has been investigated to synthesize the voltage and current in two conditions, when the input voltage of generator *V*_*s*-RMS_ is bigger than the battery voltage *V*_*s*-dc_ and vice versa. The switching period *T*_*s*_ is assumed to be 200 *μ*s in all conditions.

### 4.1. Condition I

In this condition the line-to-neutral voltage of generator *V*_*s*-RMS_ is bigger than the battery voltage *V*_*s*-dc_ and battery's charging (SOC) is equal to 50%. Thus, the power is injected from generator to the AC and DC loads. Simulation parameters for condition (I) are shown in Table 2.

In point of view of power transferring, when the battery voltage *V*_*s*-dc_ is less than the line to-neutral voltage of generator *V*_*s*-RMS_ whole load's power demand is supplied by the generator. Thus, the time interval *T*_3_ is equal to zero and voltage ratio is varied based on the value of generator's voltage.

#### 4.1.1. Voltage Synthesizing

**Figure 9:**
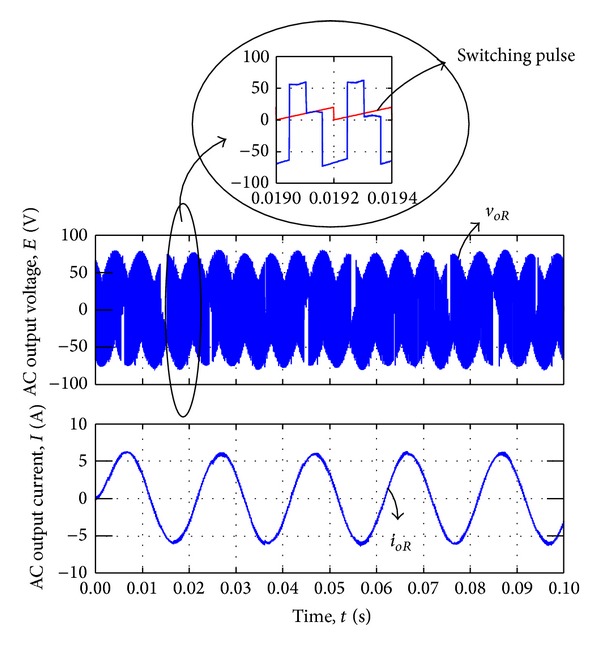
AC voltage and current output waveform by voltage synthesizing.

**Figure 10:**
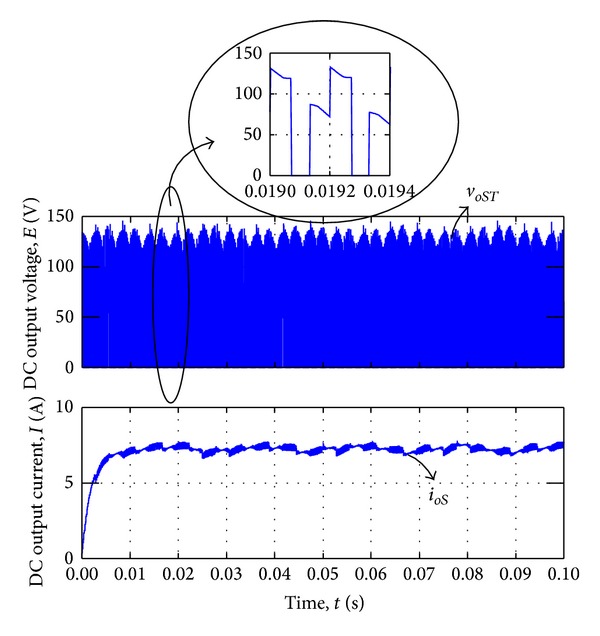
DC voltage and current output waveform by voltage synthesizing.

In voltage synthesizing situation, the slopes of triangular waveforms are constant. Therefore, the time intervals are considered to be 100 *μ*s for *T*_1_ and *T*_2_.  Figure 9 illustrates the output line-to-neutral voltage *v*_*oR*_ which is connected to the AC load and AC output current *i*_*oR*_.  Figure 10 represents the output line-to-line voltage *v*_*oST*_ which is connected to the DC loads and DC output current *i*_*oR*_. The red line in the expand graph indicated the switching pulse waveform.

**Figure 11:**
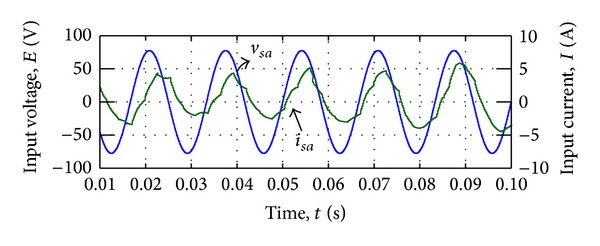
Input voltage and current waveform by voltage synthesizing.

Figure 11 shows the input phase voltage *v*_*sa*_ and filtered input current *i*_*sa*_ in steady state. The simulation results revealed that the proposed modulation method is capable of synthesizing the output voltages while the distortion on the input current is visible in Figure 11.

#### 4.1.2. Current Synthesizing

**Figure 12:**
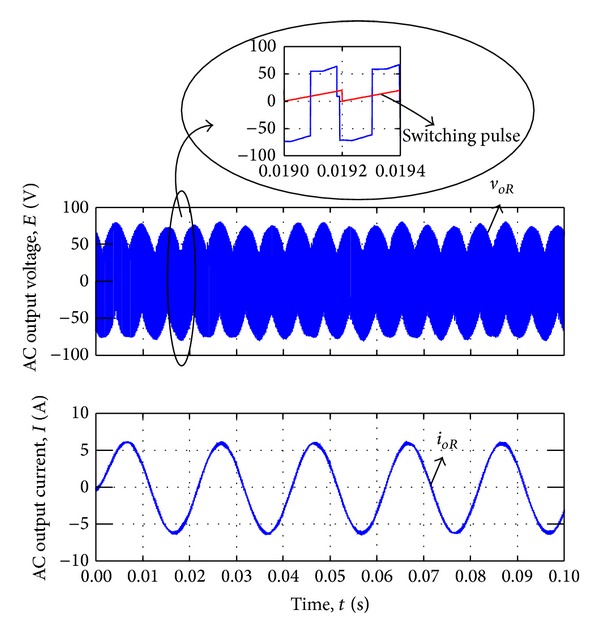
AC voltage and current output waveform by current synthesizing.

**Figure 13:**
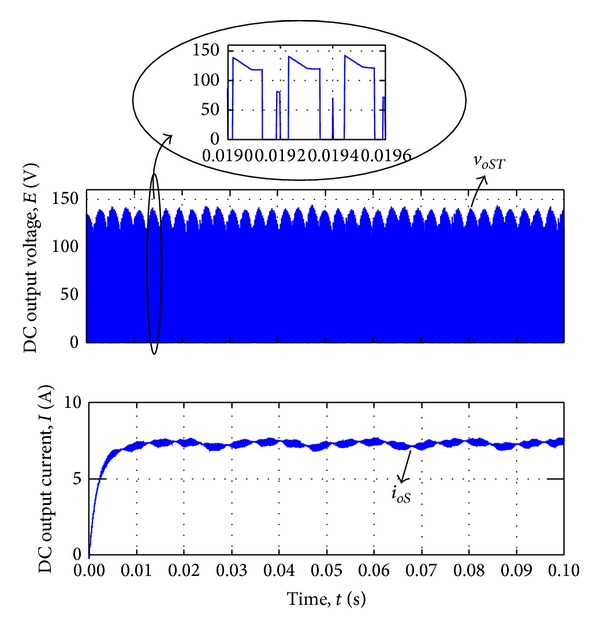
DC voltage and current output waveform by current synthesizing.

By changing the slope of the triangular carriers which is related to the value of *m*, power factor has been controlled and the sinusoidal input currents have been synthesised. Figures 12 and 13 show the output AC output voltage/current waveform and DC output voltage/current waveform, respectively.

**Figure 14:**
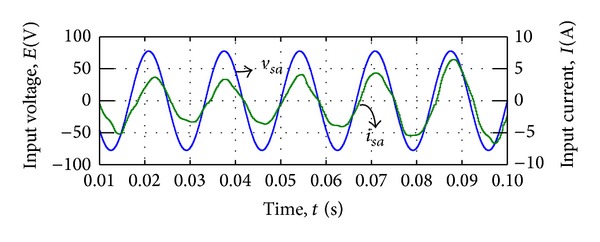
Input voltage and current waveform by current synthesizing.

Figure 14 shows the input phase voltage *v*_*sa*_ and filtered input current *i*_*sa*_ in current synthesizing mode. The simulation result in Figure 14 shows that the current input is well merged when there is no additional distortion in output voltage. The simulation result for output waveform also shows that the power factor has been controlled and unity power factor almost achieved.

### 4.2. Condition II

In the second condition when the *V*_*s*-RMS_ is less than battery voltage (*V*_*s*-dc_) and state of charge of battery's is SOC = 95%, power will be injected from generator and battery to the AC load at the same time. The switching sequence is assumed to be *T*_*s*_ = 200 *μs*. All simulation parameters are the same as parameters in Table 2, except the input voltage (line-to-neutral) *V*_*s*-RMS_, which is equal to 20 *V*_RMS_ in this condition. In condition II, the voltage ratio (*q*) is independent of the generator input voltage. The power of the generator which is transferred to the load will be determined by the differences between *V*_*s*-RMS_ and *V*_*s*-dc_.

#### 4.2.1. Voltage Synthesizing

**Figure 15:**
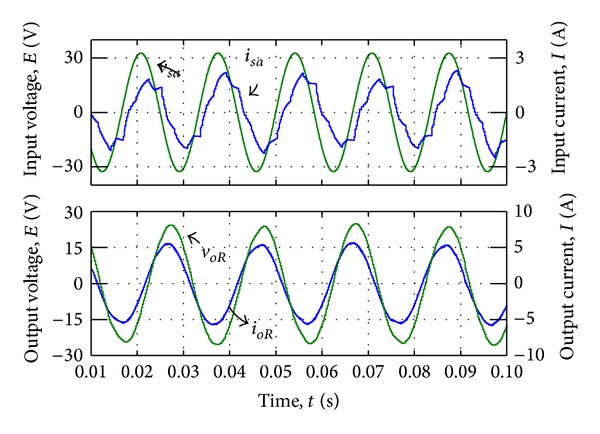
Simulation result by voltage synthesizing.

In this mode, the slopes of triangular waveforms are constant. Therefore, the time intervals are considered to be *T*_1_ = *T*_2_ = 50 *μ*s, *T*_3_ = 100 *μ*s.  Figure 15 illustrated the average output line-to-neutral voltage *v*_*oR*_ which is connected to the AC load, *R*-phase output current *i*_*oR*_, input phase voltage *v*_*sa*_, and filtered input current *i*_*sa*_. The simulation results showed that the proposed modulation method is able to well synthesize the output voltages, while the distortion on the input current is visible.

#### 4.2.2. Current Synthesising

**Figure 16:**
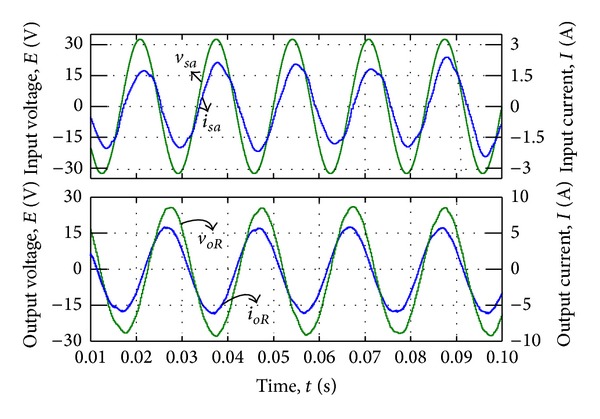
Simulation result by current synthesizing.

By changing the slope of the triangular carriers (*n* and *m*) based on Table 2, power factor has been controlled and the sinusoidal input currents have been synthesised. The simulation result in Figure 16 shows that the current input is well merged when there is no additional distortion in output voltage. The result indicated that the power factor has been controlled and unity power factor almost achieved.

### 4.3. Current Control

**Figure 17:**
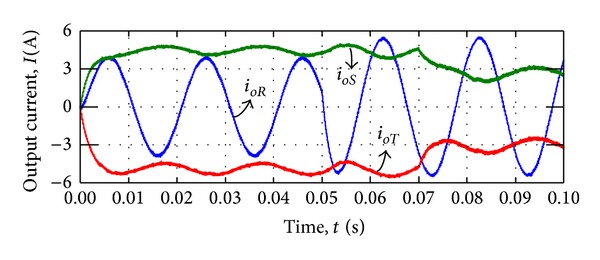
Output currents waveform during load variation.

In order to test the system stability, reference current is changed in AC and DC sides while the loads are constant.  Figure 17 shows the simulated responses of MDMC system when the current is changed in AC and DC side at *t* = 0.05 and *t* = 0.07 s, respectively.

Figure 17 shows that the current in *i*_*oS*_ and *i*_*oT*_ is constant at *t* = 0.05 regardless of the changing in *i*_*oR*_ which is increased by 0.2 pu. In addition, when the DC reference current reduced by 0.2 pu, the current in AC side remains constant at *t* = 0.07 s.

It can be clearly seen from Figure 17 that the system is able to track the variation of reference current, and current output of each terminals is completely independent of other output current terminals. Moreover, the simulation results exhibited that the undershoot/overshoot and steady-state error for output currents is acceptable for low power battery based system.

## 5. Conclusions

This study represents a structure for multidirectional matrix converter which is suitable for battery based system. This new MDMC configuration reduces the cost and size of the system. This study also presents a novel DDPWM method to control the power flow from generator and battery to the load. This new modulation method used the concept of the average voltage per switching period and a continuous carrier for multidirectional matrix converters. By applying the proper switching pattern and determining the duty ratio for each switch, the voltage of each output terminal has been well synthesized. In addition, by changing the slope of the carriers which is related to the value of *n* and *m*, power factor has been controlled and the sinusoidal input currents have been synthesised.

The feasibility of the proposed MDMC structure and EDDPWM technique has been verified by MATLAB simulation. Results of this study revealed that the proposed carrier based modulation technique can be used for the application where battery is essential. This method can easily control the power factor and merged the output voltage and input current without any lookup tables. Since this new modulation has a good flexibility and applicability, it can be effectively applied in a system with a connection between the input and output neutrals with a desired output voltage and frequency. Furthermore, battery discharging time has been increased by letting power flow from both generator and battery to the load, simultaneously. The simulation results of this study also revealed that the current response in AC and DC of the MDMC system with proposed EDDPWM method is acceptable for low power battery based systems.

## Figures and Tables

**Table 1:** Determination of *n* and *m* based on the input voltage sector.

Sector	*n*	Uniformity	*M*	Uniformity
I-2	−sin⁡⁡(*β*_*i*_ − 2*π*/3)	$\frac{\sqrt{3}}{2}D$ to *D*	(−sin⁡⁡(*β*_*i*_ + 2*π*/3))/sin⁡(*β*_*i*_ − 2*π*/3)	1–0.5 down

II-2	−sin⁡⁡(*β*_*i*_ − 2*π*/3)	$\frac{\sqrt{3}}{2}D$ to *D*	(−sin⁡⁡(*β*_*i*_))/sin⁡⁡(*β*_*i*_ − 2*π*/3)	0.5–1 up

II-1	sin⁡⁡(*β*_*i*_)	$\frac{\sqrt{3}}{2}D$ to *D*	(−sin⁡⁡(*β*_*i*_ − 2*π*/3))/sin⁡⁡(*β*_*i*_)	1–0.5 down

III-1	sin⁡⁡(*β*_*i*_)	$\frac{\sqrt{3}}{2}D$ to *D*	(−sin⁡⁡(*β*_*i*_ + 2*π*/3))/sin⁡⁡(*β*_*i*_)	0.5–1 up

III-2	−sin⁡⁡(*β*_*i*_ + 2*π*/3)	$\frac{\sqrt{3}}{2}D$ to *D*	(−sin⁡⁡(*β*_*i*_))/sin⁡⁡(*β*_*i*_ + 2*π*/3)	1–0.5 down

IV-2	−sin⁡⁡(*β*_*i*_ + 2*π*/3)	$\frac{\sqrt{3}}{2}D$ to *D*	(−sin⁡⁡(*β*_*i*_ − 2*π*/3))/sin⁡⁡(*β*_*i*_ + 2*π*/3)	0.5–1 up

IV-1	sin⁡⁡(*β*_*i*_ − 2*π*/3)	$\frac{\sqrt{3}}{2}D$ to *D*	(−sin⁡⁡(*β*_*i*_ + 2*π*/3))/sin⁡⁡(*β*_*i*_ − 2*π*/3)	1–0.5 down

V-1	sin⁡⁡(*β*_*i*_ − 2*π*/3)	$\frac{\sqrt{3}}{2}D$ to *D*	(−sin⁡⁡(*β*_*i*_))/sin⁡⁡(*β*_*i*_ − 2*π*/3)	0.5–1 up

V-2	sin⁡⁡(*β*_*i*_)	$\frac{\sqrt{3}}{2}D$ to *D*	(−sin⁡⁡(*β*_*i*_ − 2*π*/3))/sin⁡⁡(*β*_*i*_)	1–0.5 down

VI-2	sin⁡⁡(*β*_*i*_)	$\frac{\sqrt{3}}{2}D$ to *D*	(−sin⁡⁡(*β*_*i*_ + 2*π*/3))/sin⁡⁡(*β*_*i*_)	0.5–1 up

VI-1	sin⁡⁡(*β*_*i*_ + 2*π*/3)	$\frac{\sqrt{3}}{2}D$ to *D*	(−sin⁡⁡(*β*_*i*_))/sin⁡⁡(*β*_*i*_ + 2*π*/3)	1–0.5 down

I-1	sin⁡⁡(*β*_*i*_ + 2*π*/3)	$\frac{\sqrt{3}}{2}D$ to *D*	(−sin⁡⁡(*β*_*i*_ − 2*π*/3))/sin⁡⁡(*β*_*i*_ + 2*π*/3)	0.5–1 up

**Table 2:** Simulation parameter.

*R*-*L* load	*R* = 5 Ω, *L* = 10 mH
Input voltage (line-to-neutral) *V*_*s*-RMS_	56 V
Battery voltage (line-to-neutral) *V*_*s*-dc_	±48 V
Output voltage (line-to-neutral) *V*_*o*-RMS_	28 V
Voltage ratio *q*	0.5
Input frequency *f*_*s*_	60 Hz
Output frequency *f*_*o*_	50 Hz
